# Comparing the Kinetic Hydrate Inhibition Performance of Linear versus Branched Polymers

**DOI:** 10.1021/acsomega.3c09260

**Published:** 2024-03-07

**Authors:** Malcolm A. Kelland, Erik G. Dirdal, Cecilie Meidell Knutsen

**Affiliations:** Department of Chemistry, Bioscience and Environmental Engineering, Faculty of Science and Technology, University of Stavanger, N-4036 Stavanger, Norway

## Abstract

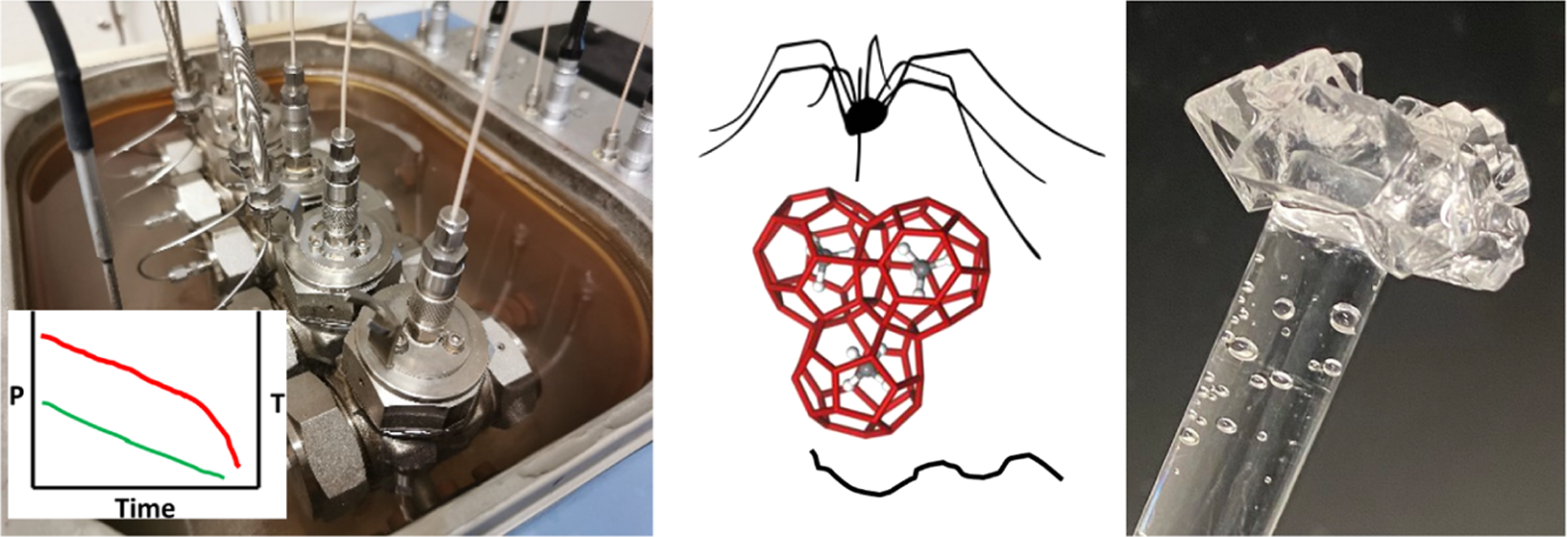

Kinetic hydrate inhibitors (KHIs) are a chemical method of preventing gas hydrate plugging of oil and gas production flow lines. The main ingredient in a KHI formulation is one or more water-soluble amphiphilic polymers. Poly(*N*-vinyl caprolactam) (PVCap) is an unbranched polymer and a well-known industrial KHI, often used as a yardstick to compare the performance of new polymers. The effect of branching PVCap on KHI performance has been investigated by polymerizing the VCap monomer in the presence of varying amounts of trimethylolpropane triacrylate, pentaerythritol tetraacrylate, or bis-pentaerythritol hexaacrylate cross-linkers to give PVCap polymers with 3, 4, and 6 branches, respectively. If the ratio of cross-linker to VCap was too high (6:1 to 8:1), gelling and/or poor water solubility was observed, giving short polymer chains and poor KHI efficacy. For higher ratios (30:1 to 60:1), it was found that the concentration of the polymer needed to give total inhibition of structure II tetrahydrofuran hydrate crystal growth could be lowered by using tribranched rather than linear PVCap. Slow constant cooling (1 °C/h) gas hydrate experiments with a synthetic natural gas in steel rocking cells at 76 bar were also carried out. A small improvement in KHI performance was observed for one of the branched PVCaps compared with a linear PVCap. Branched and linear poly(*N*-isopropylmethacrylamide) (PNIPMAm) polymers were also investigated in the gas hydrate system, but there was no benefit observed when branching this polymer class.

## 1 Introduction

Kinetic hydrate inhibitors (KHIs) are a chemical method of avoiding gas hydrate plugging, particularly in oil and gas upstream flow lines, but they can also be used in drilling and completion fluids.^1−9^ KHIs delay the hydrate formation process within the thermodynamic region for the hydrate stability. The performance is restricted by many factors, one of which is the driving force or subcooling (Δ*T*) of the system. The subcooling is the difference in the temperature between the operative temperature and the equilibrium temperature. KHIs have even been claimed to prevent hydrate formation indefinitely up to low subcooling. Several KHI mechanisms have been proposed. In most of them, the KHI polymer is assumed to interact in some way with hydrate particles (subcritical or thermodynamically stable crystals) to prevent or inhibit further growth.^10^ Certain KHI polymers have also been shown to inhibit the growth of preformed gas hydrates up to certain subcoolings.^11^

**Figure 1:**
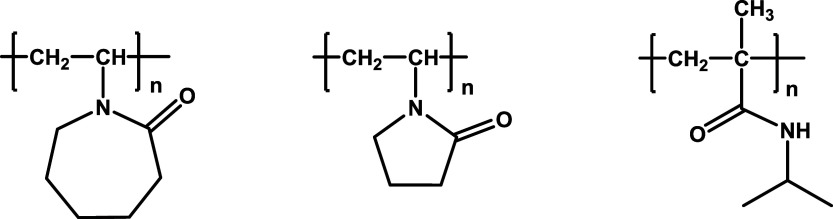
Left to right, poly(*N*-vinyl caprolactam) (PVCap), poly(*N*-vinylpyrrolidone) (PVP), and poly(*N*-isopropylmethacrylamide) (PNIPMAm).

KHIs are pumped into the hydrate-forming fluids before they cool and pass the phase boundary for the thermodynamic stability of gas hydrates. The main component in liquid KHI formulations is one or more water-soluble oligomers or polymers that contain peripheral amphiphilic groups. These groups have hydrophilic parts that have strong hydrogen bonding properties such as amide, imide, or amine oxide, although many other functional groups have been investigated.^12−15^ Well-known examples of KHI polymers include homo- or copolymers of *N*-vinyl caprolactam (VCap), *N*-vinylpyrrolidone (VP), and *N*-isopropylmethacrylamide (NIPMAm) (Figure 1). The homopolymer of VCap, PVCap, is a powerful KHI and is often used as a standard to gauge the performance of other polymers.^16^ PVCap is a thermoresponsive polymer in aqueous solution, meaning that it becomes insoluble above a specific temperature.^17,18^ For PVCap, this lies at about 30–45 °C depending on the polymerization method, which can affect parameters such as molecular weight, endcapping, tacticity, and branching.

**Figure 2:**
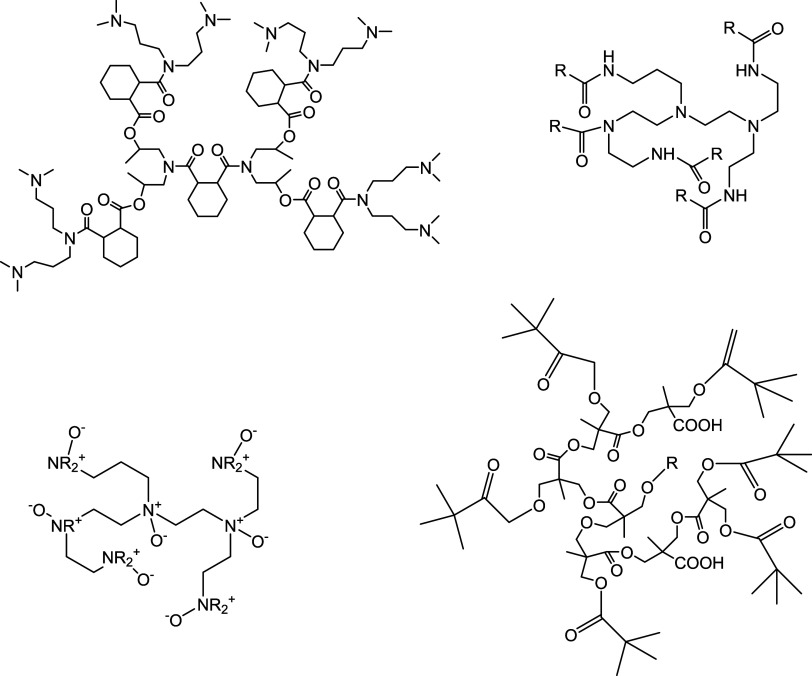
Examples of hyperbranched polymeric KHIs. Clockwise from top left: poly(ester amide)s, polyethylenimine acylamides, polyesters, and alkylated polyethylenimine oxides.

The structure of polymers can be tailored in many different ways, and there are many ways of controlling the architecture even using classical radical polymerization techniques.^19^ Branching is one type of polymer architecture, and this can take several forms including hyperbranching or dendrimeric.^20−23^ Branched polymers have been investigated for several oilfield production flow line applications. This includes wax, corrosion, and scale inhibitors.^24−29^ Within the field of KHIs, only a limited amount of work has been done on branched polymers with even less on systematic studies comparing linear to branched polymers with the same monomer units.^30^ Shell has investigated hyperbranched poly(ester amide)s as KHIs as part of a class of commercial polymers (Figure 2).^31−33^ However, they were not compared to linear polymers with the same functionality and regularity of amphiphilic groups. Hyperbranched polyesters with peripheral hydrophobic groups such as t-butyl have also been claimed as KHIs (Figure 2).^34^

Hyperbranched polymers of polyethylenimine (PEI) have been reported and compared to linear polymers (Figure 2).^35^ Butylated linear PEI amine oxides were shown to be excellent tetrahydrofuran (THF) structure II (sII) hydrate crystal growth inhibitors, superior to hyperbranched PEI amine oxide derivatives, also with butyl groups. However, the linear PEI amine oxides were poorer gas hydrate KHIs than the equivalent hyperbranched PEI derivatives. The reason was proposed to be related to the availability of primary amine groups in HPEI or oligomeric ethyleneamines. Acylamide derivatives of PEI polymers were also investigated.^36^ Acylamides based on linear PEI gave better performance than those based on hyperbranched PEI. These studies with amine oxide and acylamide derivatives of PEI confirmed that the skeletal polymer architecture can affect hydrate inhibition. Another report related to PEI proposes branched KHIs based on the reaction of pyroglutamic acid with hyperbranched PEI.^37^ Baker Hughes has reported and commercialized branched *N*-vinyl lactam polymers in which a branched ester-based chain transfer agent with thiol end groups is first synthesized.^38^ A typical example uses various ratios of thioglycolic acid, sorbitol, and citric acid. This can then be used in a radical polymerization process with *N*-vinyl lactam monomers such as VCap or *N*-vinyl pyrrolidinone and glycerol dimethacrylate to form the branched polymers.

Poly(*N*-isopropylacrylamide) (PNIPAM) polymers synthesized using reversible addition–fragmentation chain-transfer (RAFT) polymerization have also been studied in both linear and branched architectures.^39^ A linear PNIPAM polymer delayed gas hydrate nucleation to a similar extent to linear PVP and PVCap, but the branched PNIPAm polymer gave improved gas hydrate crystal growth inhibition. A branched polymer showed better performance than that of the equivalent linear polymer in terms of hydrate fraction and resistance to flow. Other branched natural polymers, such as xanthan, have also been studied as KHIs. They all have very poor KHI performance when tested alone and under typical field conditions. They may be affecting the gas mass transport by increasing the viscosity, which will slow the rate of hydrate formation.^40−43^ Even grafting acrylamide or other purely hydrophilic monomers does not improve the performance significantly for gas hydrate systems because you need amphiphilic groups such as those found in VCap or NIPMAm to be added.^44^ Branched polycitramides were shown to give good KHI performance but only when suitably large (C3–C6) alkyl groups were attached to the amide groups.^45^

**Figure 3:**
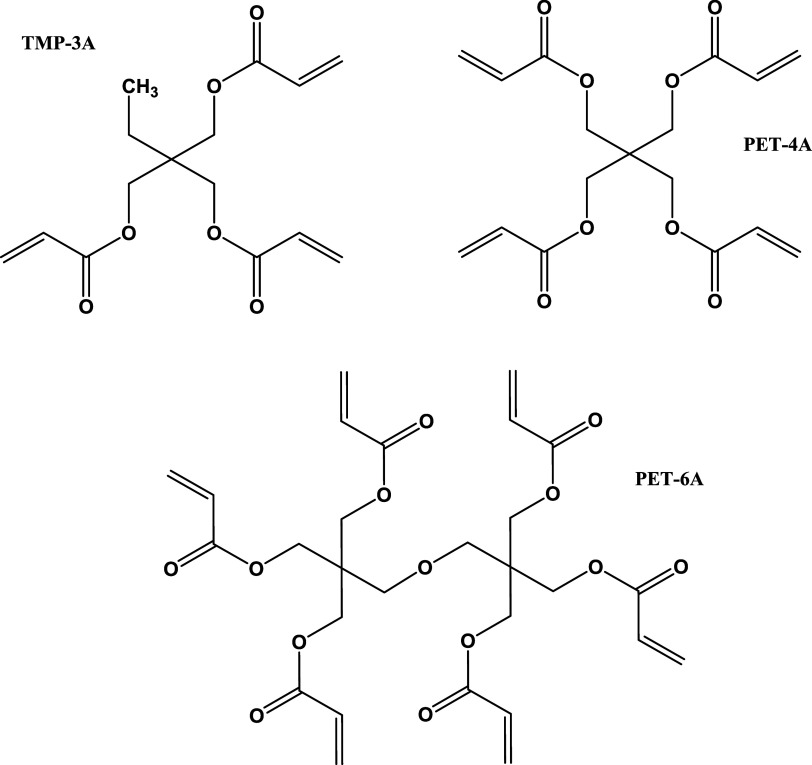
Structures of the cross-linkers trimethylolpropane triacrylate (TMP-3A), pentaerythritol tetraacrylate (PET-4A), and bis-pentaerythritol hexaacrylate (PET-6A).

Diacrylate cross-linkers have been proposed for use in KHI polymers.^46^ Trimethylolpropane triacrylate (TMP-3A), pentaerythritol tetraacrylate (PET-4A), and bis-pentaerythritol hexaacrylate (PET-6A) are well-known polymer cross-linkers used in several industrial applications that can give branched polymers at the correct monomer:cross-linker ratio (Figure 3).^47,48^ However, to the best of our knowledge, these cross-linkers have not been used to make branched KHI polymers. Here, we report the first study of branched versus linear PVCap using these three cross-linkers to form polymers with essentially 3, 4, and 6 branches. Experiments were conducted with tetrahydrofuran hydrate, which forms sII hydrate at atmospheric pressure, as well as a natural gas mixture to give sII hydrate as the most thermodynamically stable phase.

## 2 Experimental Methods

### 2.1 Synthesis and Characterization

**Figure 4:**
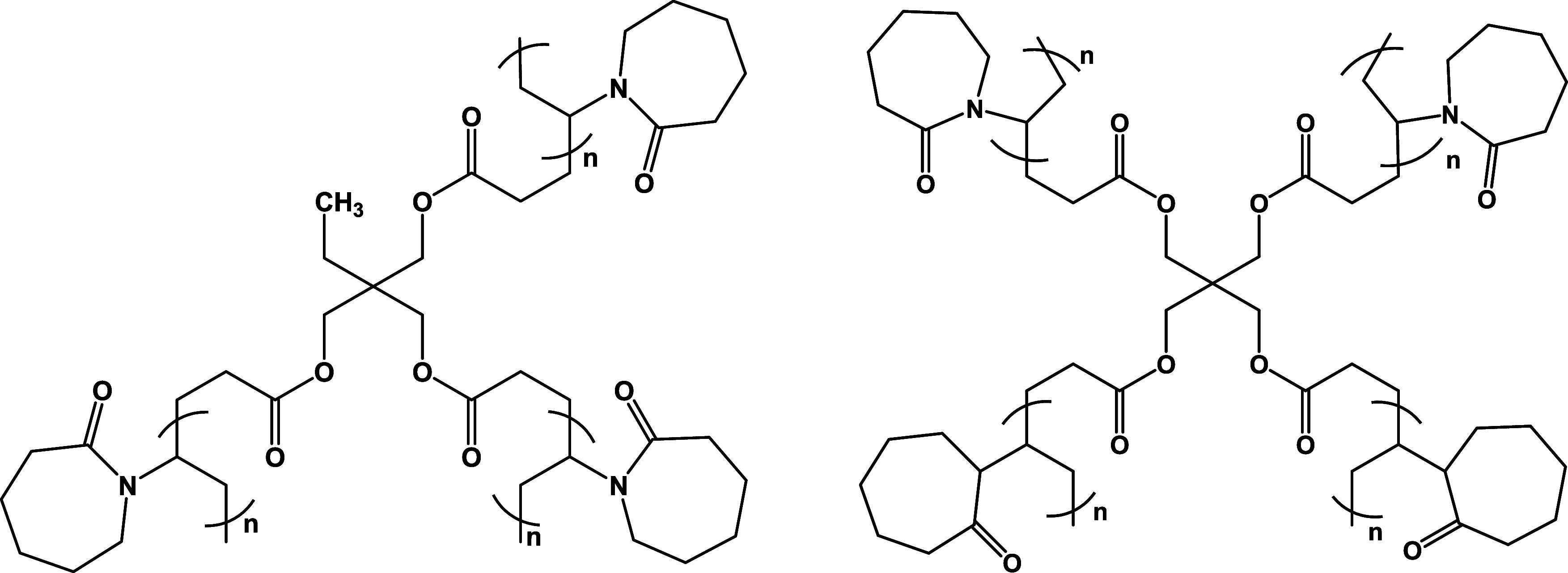
Idealized structures of TMP-3A-PVCap (left) and PET-4A-PVCap (right).

PVCap (41.1 wt % in monoethylene glycol) was supplied by BASF as Luvicap EG. The glycol solvent was removed by repeated precipitation above the cloud point as a solution in deionized water. ^1^H NMR spectroscopy in D_2_O showed pure PVCap without any glycol. The molecular weight (*M*_n_) was measured by size exclusion chromatography (SEC) as 2400 g/mol (details of the method given at the end of the paragraph). All other chemicals were supplied by Merck or Avantor. Polymerization of VCap or NIPMAm alone or with the three cross-linkers TMP-3A, PET-4A, and PET-6A was carried out using the same procedure but at different ratios. A typical synthesis is given for TMP-3A-PVCap 6:1 (Figure 4) as follows: The VCap monomer (4.0 g, 0.0288 mol), TMP-3A (1.42 g 0.0048 mol), 2,2′-bis-azo-isobutyronitrile (AIBN) (0.04 g), 2-propanol (10 mL), and a magnetic stirrer bar were placed in a closed Schlenk tube. Air was replaced by dinitrogen, and the contents were stirred and heated to 70 °C for 18–20 h. ^1^H NMR spectroscopic analysis indicated no vinylic protons as proof that all VCap monomers had been polymerized. All products were made as approximately 30% solutions of the polymer in iPrOH and are listed in Table 1. Some polymerizations led to gelled products, which are also indicated in Table 1. Another example of a branched VCap-based polymer is given for PET-4A-PVCap, made by polymerizing VCap with PET-4A (Figure 4). Polymer molecular weight analysis for all polymers was carried out by size exclusion chromatography (SEC) using DMF solvent at 0.6 mL/min and 40 °C, using polystyrene standards. The apparatus used was a JASCO Chem NAV size exclusion chromatography system. This system was equipped with PU-2080, AS-2055, CO-2065 RI-2031, and two commercial columns (TSKgel SuperH4000 and TSKgel GMHXL).

**Table 1:** Composition of the Synthetic Natural Gas Mixture (SNG)

component	mol %
nitrogen	0.11
*n*-butane	0.72
isobutane	1.65
propane	5.00
CO_2_	1.82
ethane	10.3
methane	80.4

### 2.2 Cloud Point

A 2500 ppm solution of polymer in deionized water was heated until cloudiness appeared. A rough cloud point temperature (Tcl) was noted. The solution was cooled below Tcl until clear. Then, the solution was reheated at about 5 °C/min until the solution went cloudy again. This gave the exact Tcl that was recorded. The process was repeated for checking the reproducibility. For some polymers, there was a small amount of insoluble material at all temperatures of 0–100 °C. In most cases, a cloud point could still be obtained from the remaining solution.

### 2.3 THF Hydrate Test Method

The equipment and general method for studying the inhibition of THF hydrate crystal growth have been reported previously.^33,49−55^ The solution for making THF hydrate crystals consists of NaCl (26.28 g) and THF (99.9%, 170 g) mixed with distilled water to give a final volume of 900 mL. This blend gives a stoichiometrically correct molar composition for making structure II (sII) THF hydrate, THF·17H_2_O. The sodium chloride drops the THF hydrate formation equilibrium temperature to 3.3 °C. The salt addition allows for testing at temperatures below the ice point (0 °C) without giving too high a subcooling. The test temperature must be kept below 0 °C to avoid the ice melting in the glass tube, which is placed in the beakers. The complete test procedure is as follows:

1. 80 mL of the aqueous THF/sodium chloride solution is placed in an unscratched 100 mL glass beaker.
2. The test chemical is dissolved in this solution to give the desired concentration. As an example, 0.4 g of polymer in 80 mL of aqueous solution will give a 0.5 wt % (5000 ppm) solution of the polymer.
3. The beaker with solution is placed in a stirred cooling bath preset to a set temperature, e.g., −0.5 °C, which represents about 3.8 °C subcooling.
4. The solution is briefly stirred every 5 min for 20 min with a plastic rod.
5. A hollow glass tube with an inner diameter of 3 mm was filled at the end with ice crystals kept at −20 °C.
6. The glass tube is placed in the cooled test solution. The ice crystals at the end of the tube initiate THF hydrate formation.
7. THF hydrate crystals were allowed to grow at the end of the glass tube for 60 min.
8. The glass tube was removed. If any THF hydrate crystals were present on the tube tip, the concentration of the polymer was increased, usually in 250 ppm increments, and the test was repeated. The concentration of the polymer at which no THF hydrate was formed in 1 h was determined. The shape and morphology of the crystals in the beaker (if any) and on the end of the glass tube were also recorded. With no additive, pyramidal crystals are formed.^33,49−55^

### 2.4 Gas Hydrate Testing Method

#### 2.4.1 Slow Constant Cooling (SCC) KHI Experimental Test Procedure

The equipment used was five parallel 40 mL stainless-steel cells placed in a water bath with a temperature controller. These cells were rocked at 20 rocks/min while cooling. The rocking equipment was supplied by PSL Systemtechnik, Germany, with the cells supplied by Swafas, Norway. Each steel cell contains a stainless-steel ball, which agitates the fluids during rocking. Each cell is equipped with a pressure and temperature sensor. Each cell was pressurized separately with a synthetic natural gas (SNG). The composition is given in Table 1. The SNG preferentially forms structure II (sII) gas hydrates as the most thermodynamically stable phase.

KHI polymers were evaluated for performance by the slow constant cooling (SCC) experimental method and is summarized as follows:^49^

1. Polymers were dissolved to the desired concentration in deionized water usually 1 day in advance of the test to bring the solution to equilibrium.
2. 20 mL of test solution was added to each of the five cells and the cells were sealed and placed in the cooling bath.
3. After removing the air from the cells with vacuum pumping, the cells were pressurized with 76 bar SNG.
4. The five cells were rocked at a rate of 20 rocks per minute with an angle of 40°, while being cooled at 1.0 °C/h from 20.5 to 2.0 °C.

The hydrate equilibrium temperature (*T*_eq_) at 76 bar SNG was found previously to be 20.2 ± 0.05 °C warming at 0.025 °C/h for the last 3–4 °C. This was determined by standard laboratory dissociation experiments and correlates well with calculations done using PVTSim software (Calsep, Denmark).^56^

**Figure 5:**
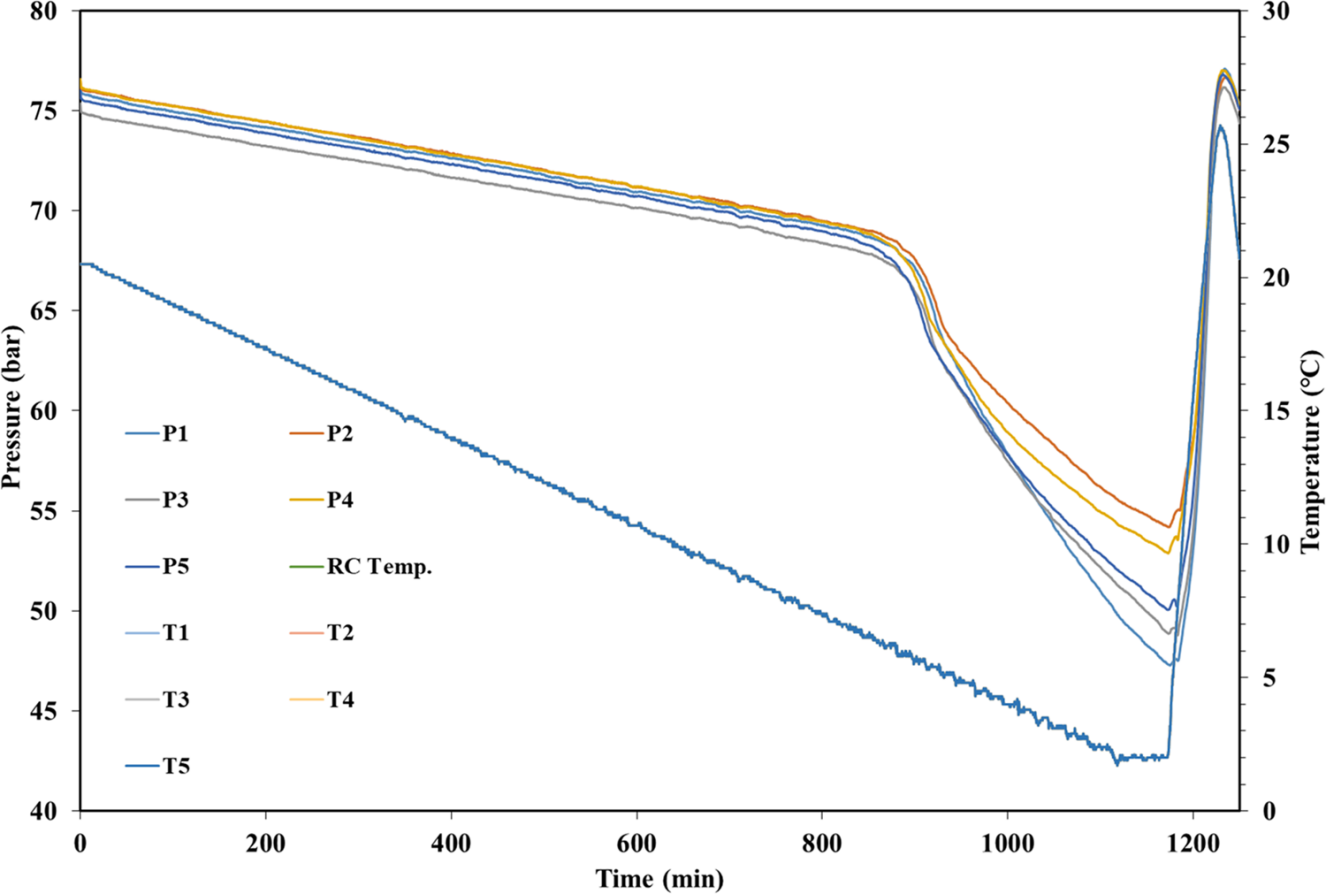
Summary graph of a typical slow constant cooling in all five rocking cells. The temperature of cell 5 (T5) is shown only for clarity.

**Figure 6:**
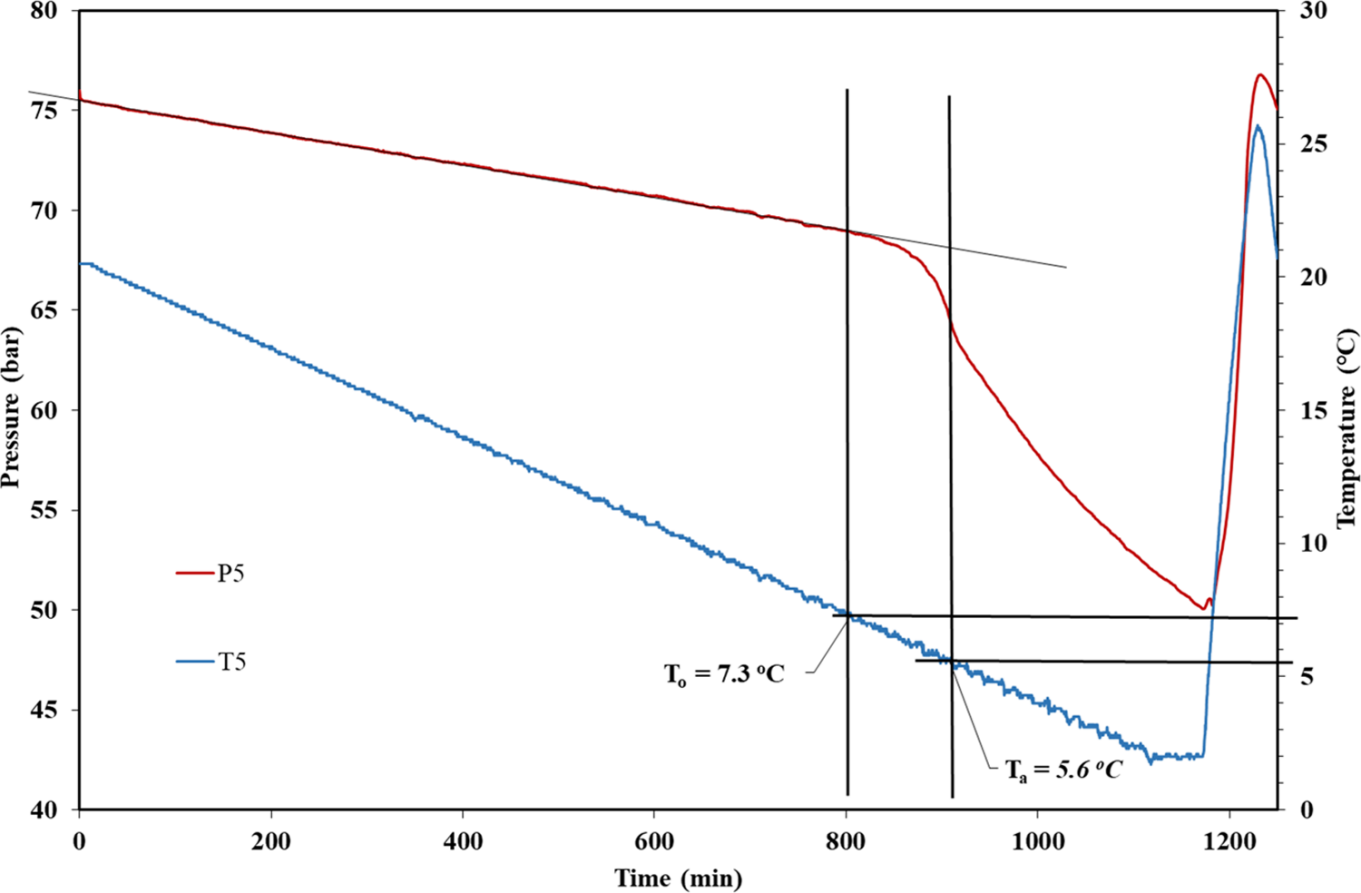
Determination of *T*_o_ and *T*_a_ values for one rocking cell constant cooling experiment.

During cooling in SCC experiments, a linear pressure decrease occurs in the closed system until the first detected onset of hydrate formation (*T*_o_) when the pressure drops faster due to consumption of SNG. The start of nucleation may possibly happen earlier than *T*_o_. *T*_a_ is taken as the temperature when the pressure decrease is at its steepest, i.e., when the hydrate formation is at its fastest. Figure 5 shows data for all five cells and illustrates the level of reproducibility. In the single test result in Figure 6, *T*_o_ is determined as 7.3 °C and *T*_a_ is determined as 5.6 °C. The standard deviation (assuming a normal distribution) for a set of *T*_o_ or *T*_a_ values is no more than 0.6 °C and usually less than 0.3 °C. The scattering still allows for a rough ranking of the performance of the KHI samples as long as sufficient tests are carried out for a statistically significant difference using a *t* test. Depending on the variation in average *T*_o_ between samples, 5–10 tests are usually sufficient to get a significant difference at the 95% confidence level (*p* < 0.05).^57^ Gas hydrate formation is a stochastic process. Results for four sets of tests in five rocking cells for one polymer using the same test method as this study were recently reported, demonstrating the reproducibility.^58^ Thus, five tests allow for ranking of polymers as long as the average *T*_o_ value for two different polymers is sufficiently different, as warranted by the p-value obtained in a *t* test. In addition, the rocking cell SCC test has been shown to be comparable to the autoclave isothermal test in its ability to screen and rank KHI performances.^10,59,60^

## 3 Results and Discussion

### 3.1 Polymer Synthesis and Characterization

The cores of the branched polymers are made from the known cross-linkers TMP-3A, PET-4A, and PET-6A. Polymerization of monomers onto these multivinylic molecules gives the idealized branching, illustrated for TMP-3A-PVCap and PET-4A-PVCap in Figure 4.^61,62^ A summary of the polymers used in this study is given in Table 2.

**Table 2:** Polymers Prepared in This Study and Molecular Weight and Cloud Point (Tcl) Data in Water at 2500 ppm

polymer	molecular weight (*M*_n_) (g/mol)	PDI	cloud point (Tcl) (°C)
PVCap, linear	2400	1.80	39
TMP-3A-PVCap 6:1	1800	2.60	mostly insoluble
TMP-3A-PVCap 30:1	1200	3.61	40
TMP-3A-PVCap 60:1	1500	4.28	38
PET-4A-PVCap 8:1 (gel)	1400	6.53	41
PET-4A-PVCap 20:1	2300	4.74	39
PET-4A-PVCap 40:1	1800	3.78	39
PET-4A-PVCap 80:1	2900	4.10	38
PET-6A-PVCap 8:1 (gel)	1500	3.82	36
PET-6A-PVCap 40:1	2800	4.04	34
PNIPMAm, linear, 1.3k	1300	1.93	39
PNIPMAm, linear 24.4k	24,400	1.90	32
PET-4A-PNIPMAm 8:1	6300	1.59	cloudy at 0–100 °C
PET-4A-PNIPMAm 40:1	11,000	1.70	30
PET-6A-PNIPMAm 8:1	7300	1.70	cloudy at 0–100 °C
PET-6A-PNIPMAm 40:1	11,000	1.83	35

The gel permeation chromatography (GPC) data are difficult to interpret for branched polymers because the method is size exclusion and does not take into account the volume of a polymer, especially branched polymers. GPC/SEC separates only based on the size of the molecule in solution. It is well known that molecular weight distribution has a strong effect on the KHI performance with the majority of the polymer being low molecular weight (600–5000 g/mol) being the best for gas hydrate inhibition.^63,64^ In this study, we did manage to make polymers that gave low and similar molecular weight, which allowed for performance comparison as KHIs on THF hydrate and gas hydrate inhibition. Data for PVCap are for a commercial sample, and PNIPMAm samples were made by the literature method.^65^ We had difficulty making a nonbranched PNIPMAm with Mw values near those of the branched PNIPMAm polymers without using a chain transfer agent. Therefore, using only the AIBN initiator, we prepared a low*-M*_n_ (1300 g/mol) and higher *M*_n_ (24,400 g/mol) PNIPMAm.

As the molar percentage of the cross-linker compared to the comonomer increases, the amount of cross-linking increases. This often has the effect of gel formation (hydrogelling) of the resulting polymer if the polymer concentration is high and the molecular weight (size of the polymer in SEC) is small.^66,67^ This occurred for some of the branched PVCap polymers made in this study, particularly those with a low VCap:cross-linker ratio. Specifically, this occurred for PET-4A-PVCap 8:1 and PET-6A-PVCap 8:1, both of which have low molecular weights. However, a cloud point could still be obtained by dissolving the gel in water. Gelling due to cross-linking may have been reduced by polymerization under very diluted conditions, but this is not viable for commercial application. Concentrated polymer solutions make the logistics of transporting and injecting KHI more economical. For the PNIPMAm branched polymers, NIPMAm polymerizes more rapidly than VCap. This led to comparably higher molecular weight polymers compared to the equivalent branched PVCap product. The majority of these PNIPMAm products were therefore not gelled, in contrast to the low-ratio branched PVCap polymers. However, a small amount (<5%) of two products at 8:1 ratio PET-4A-PNIPMAm and PET-6A-PNIPMAm branched polymers was difficult to dissolve, which made it difficult to determine a cloud point. For the other polymers in Table 3, the cloud points of the branched VCap- and NIPMAm-based polymers did not differ much from the equivalent linear polymers.

**Table 3:** THF Hydrate Crystal Growth Observed at −0.3 °C^a^

polymer	molecular weight (*M*_n_) g/mol (PDI)	MPC (ppm)
PVCap, linear	2400	3000
TMP-3A-PVCap 6:1	1800	mostly insoluble
TMP-3A-PVCap 30:1	1200	3100
TMP-3A-PVCap 60:1	1500	2500
PET-4A-PVCap 8:1	1400	partially soluble
PET-4A-PVCap 20:1	2300	partially soluble
PET-4A-PVCap 40:1	1800	3500
PET-4A-PVCap 80:1	2900	3500
PET-6A-PVCap 8:1 (gel)	1500	4500^b^
PET-6A-PVCap 40:1	2800	>5000^b^

a MPC = minimum polymer concentration for complete crystal growth inhibition.

b Not fully soluble.

### 3.2 THF Hydrate Crystal Growth Inhibition Results

**Figure 7:**
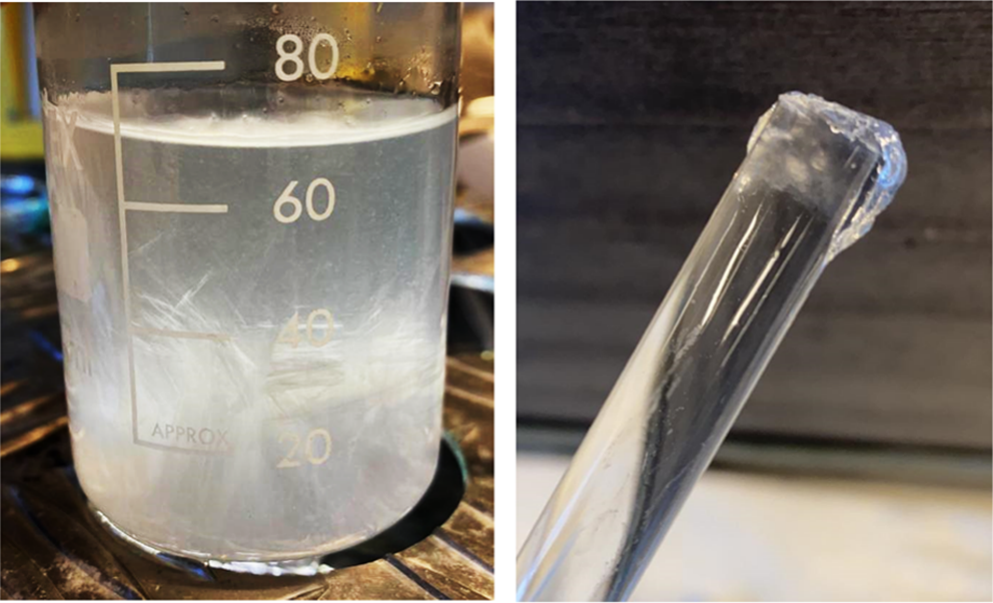
Left, THF hydrate crystal growth in the form of plates throughout the whole solution using 3000 ppm PET-6A-PVCap 40. Right, a small amount of THF hydrate crystals with TMP-3A-PVCap 60:1 at 2100 ppm, a concentration 400 ppm less than that of the MPC.

Table 3 summarizes the results of the THF hydrate crystal growth inhibition studies. The NIPMAm polymers were not investigated as they have been shown previously to be much less active compared to VCap polymers in inhibiting THF hydrate crystal growth.^2,68^ Therefore, it would be difficult to compare their activity at typical applied KHI polymer concentrations of 1000–10,000 ppm. The minimum polymer concentration (MPC) to completely inhibit THF hydrate crystal growth was determined to be 100 ppm. Some of the polymers were not fully soluble in the THF/NaCl aqueous solution. This was most apparent for the polymers with a low VCap/cross-linker ratio because the cross-linker is less hydrophilic than the VCap units and dominates more at the low ratio. There may also be a cononsolvency issue for some of these polymers in the THF/water mixture.^69−71^ Apart from the normal pyramidal crystals which are seen for no additive or low concentrations (<500 ppm) of the polymer, three other observations were made regarding THF hydrate growth. In increasing concentration of the polymer, we observed the following: (1) thin plates formed in the whole of the beaker, (2) a small amount of THF hydrate growth was observed on the glass tube tip, as thin plates, and (3) no growth was observed at or above the MPC. Examples of effects (1) and (2) are shown in Figure 7.

Linear PVCap (*M*_n_ 2400 g/mol) gave complete inhibition of THF hydrate at an MPC of 3000 ppm. This fits well with previously reported data at the same subcooling in which a PVCap with a slightly lower *M*_n_ value of 2000 g/mol gave an MPC of 3200 ppm.^55^ It is important to note that in this earlier study, the MPC decreased down to 2700 ppm as the molecular weight of PVCap increased from 2000 to 20000 g/mol. This was proposed to be due to the strength of the interaction of the polymer with the hydrate surface increasing as the number of monomer units in the polymer increases.

**Figure 8:**
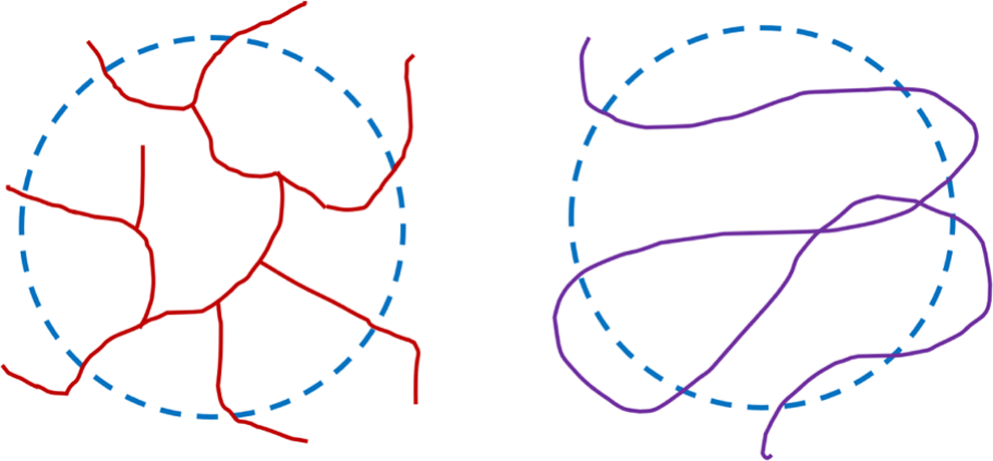
Schematic of the volume of a branched or hyperbranched polymer (left) and a linear polymer, illustrating the difference in density.

Concerning the acrylate-branched PVCaps, the only polymer that gave an MPC less than 3000 ppm (i.e., better than linear PVCap) was TMP-3A-PVCap 60:1, which gave an MPC of 2500 ppm. This result can be rationalized from the length of the polymer chains. Linear PVCap has an *M*_n_ value of 2400 g/mol, which means that there are on average roughly 17 monomer units per chain. This is a reliable molecular weight. For the tribranched polymer TMP-3A-PVCap 60:1, the *M*_n_ value from SEC was 1500 g/mol. However, as discussed earlier, the *M*_n_ values of the branched polymers are less reliable. This can be seen from the schematic in Figure 8 where the density is higher for the branched polymer. This may be counterintuitive considering that branching typically results in the final product being more flexible or less dense, but on a molecular level, a branch point increases the amount of mass in a given volume, thus increasing the molecular density. Therefore, given the ratio of 60:1 between the triacrylate cross-linker and VCap, we would expect to get chains of about 20 monomer units per acrylate. If the three chains of about 20 monomer units each are all interacting with the THF hydrate surface TMP-3A-PVCap 60:1, it would be expected to give better inhibition than a single arm of linear PVCap with 17 monomer units. This analysis also fits with TMP-3A-PVCap 30:1, which gave an MPC very similar to that of linear PVCap. TMP-3A-PVCap 30:1 has 3 chains of roughly 10 monomer units. The longest straight chain would be about 20 units, similar to linear PVCap.

The tetra- and hexabranched PVCap samples made using PET-4A and PET-6A, respectively, did not perform as well as linear PVCap. Tests on all polymers were repeated several times and shown to be reproducible. The PET-4A-PVCap 40:1 and PET-4A-PVCap 80:1 polymers were fully soluble and gave MPC values of 3500 ppm compared to the 3000 ppm for linear PVCap. We suspect that there is more cross-linking using the tetra- and hexaacrylate cross-linkers than with the triacrylate. This is reflected in the solubility of the polymers. Even PET-4A-PVCap 20:1 was not fully soluble, probably due to sufficient cross-linking to give some gelled polymer. None of the hexaacrylate cross-linked polymers were fully soluble, which is why they gave high MPC values (Table 4).

**Table 4:** Gas Hydrate Slow Constant Cooling Test Results with 2500 ppm Aqueous Polymer Solution

polymer	*T*_o_ (av.) (^o^C)	*T*_a_ (av.) (^o^C)	solubility at 20 ^o^C and foaminess
no additive	17.1	16.9	
PVCap, linear	8.4	6.5	soluble
TMP-3A-PVCap 6:1	11.4	10.5	many dispersed particles, some foam
TMP-3A-PVCap 30:1	8.2	7.6	soluble, foamy
TMP-3A-PVCap 60:1	8.0	7.3	soluble, foamy
PET-4A-PVCap 8:1 (gel)	8.4	7.2	opaque
PET-4A-PVCap 20:1	8.1	6.7	soluble
PET-4A-PVCap 40:1	7.4	6.1	soluble
PET-4A-PVCap 80:1	8.0	6.8	soluble
PET-6A-PVCap 8:1 (gel)	8.8	6.0	mildly cloudy even below Tcl
PET-6A-PVCap 40:1	8.9	8.7	<5% insoluble
PNIPMAm linear 1.3k	8.4	6.5	soluble
PNIPMAm linear 24.4k^76^	10.0	9.6	soluble
PNIPMAm linear 22.4k^77^	9.3	9.0	soluble
PET-4A-PNIPMAm 8:1	10.6	10.2	not fully soluble, some foam
PET-4A-PNIPMAm 40:1	9.1	8.6	soluble
PET-6A-PNIPMAm 8:1	10.4	9.9	not fully soluble, some foam
PET-6A-PNIPMAm 40:1	9.3	8.5	soluble

The conclusion from the THF hydrate studies was that enhanced crystal growth inhibition compared to linear PVCap was possible with mild cross-linking using a triacrylate (PET-3A), but the chains of VCap monomer units are required to be long to avoid gelling and poor solubility.

### 3.3 Gas Hydrate KHI Screening Tests

Table 4 summarizes the gas hydrate KHI performance screening results from slow constant cooling (1 °C/h) tests with an SNG initially at 76 bar. To confirm the trend in results with branched PVCap polymers, we also carried out KHI performance screening SCC tests with branched and linear PNIPMAm. Solubility and foam issues are also noted in Table 4. As with the THF hydrate tests, some polymers were not fully soluble in deionized water at 2500 ppm. This was probably due to too much cross-linking of some of the polymer chains. If a polymer was judged visually to be nearly fully soluble, then, it was tested for KHI performance. However, care must be taken in interpreting the performance since any solid deposits in the cell may have hindered heteronucleation, possibly giving an artificially lower *T*_o_ value. Foam was observed to be formed for some of the polymers in Table 4 and was the most strong when releasing the gas pressure in the cells. Degassing had to be done more slowly to avoid foam up the steel lines. When the cells were opened, the foaminess was visually seen.

All polymers gave a good KHI effect, significantly better than that of water alone, which is the first entry in Table 4. The performance (*T*_o_ and *T*_a_ values) of the linear versions of PVCap and PNIPMAm was within the same range as reported previously.^72,73^ For both the VCap and NIPMAM branched polymers, polymers with short chains (i.e., where the ratio of VCap to cross-linker is 6:1 or 8:1) were still able to give a reasonable KHI effect if fully soluble. Thus, PET-4A-PVCap 8:1 gave an average *T*_o_ of 8.4 °C. Other polymers with short chains were only partially soluble, making it difficult to gauge the true effect of the soluble fraction. Linear polymers with short chains have previously been shown to give good KHI performance previously. Monomers or dimers behave poorly, as the chains are too short for good KHI efficacy. One early study on PVCap found that the highest subcooling performance was obtained with a polymer molecular weight of 900 g/mol, and the next best PVCap was 1300 g/mol with less and less performance as the molecular weight increased.^74,75^ Only the number-average molecular weight (*M*_n_) was reported. A value of 900 g/mol represents about 6–7 monomer units.

For the other branched VCap polymers with longer chains (cross-linker:monomer ratio of 30:1 to 80:1), only one polymer, PET-4A-PVCap 40:1, gave a significantly lower average *T*_o_ value than that of linear PVCap. The average *T*_o_ value was 7.4 °C compared to the 8.4 °C for linear PVCap. The average *T*_a_ values of the branched and linear polymers, 6.2 and 6.5 °C, respectively, were not significantly different. For PNIPMAM, both branched polymers gave similar performances (average *T*_o_ = 9.1 and 9.3 °C) compared to two higher molecular weight linear versions (average *T*_o_ = 10.0 and 9.3 °C). The two linear PNIPMAm polymers made in house by identical methods and with molecular weights of 24400 and 22400 g/mol are given in Table 4 to illustrate the reproducibility of the synthesis and KHI performance. The low-molecular version PNPMAm 1.3k (*M*_n_ = 1300 g/mol) gave better KHI performance than that of any other NIPMAM polymer with an average *T*_o_ of 8.4 °C. In summary, it is possible to achieve a small improvement in KHI performance by branching the VCap-based polymer, although an even lower molecular weight linear PVCap may have worked better. For the NIPMAm-based polymers, branching did not provide a polymer with better performance than that of the low-molecular weight polymer.

## 4 Conclusions

A series of branched PVCap and PNIPMAm polymers were made using tri-, tetra-, and hexaacrylate cross-linkers. The measured *M*_n_ values by GPC were low for branched PVCaps (<3000 g/mol) and 6000–11,000 g/mol for PNIPMAms. Poor water solubility and/or polymer gelling was observed if the ratio of VCap or NIPMAm to cross-linker was low (6:1 to 8:1). A few polymers had limited solubility that this could be challenging to make them effective KHIs in field conditions.

THF hydrate crystal growth experiments were carried out for PVCap polymers. Complete crystal growth inhibition (MPC) was possible at 2500 ppm for TMP-3A-PVCap 60:1 compared to 3000 ppm for linear PVCap. This branched polymer was the only polymer that performed better than linear PVCap. Thus, mild cross-linking was advantageous with sufficiently long chains of VCap monomer units to avoid gelling and poor solubility.

Gas hydrate constant cooling tests with an SNG showed only a marginal improvement in KHI performance (primarily nucleation inhibition) by branching the VCap-based polymer, and this only occurred for PET-4A-PVCap 40:1. The branched PVCap that performed best to inhibit THF hydrate crystal growth was not the best polymer to inhibit gas hydrate growth. However, the gas hydrate also involves nucleation inhibition. For the polyNIPMAm, branching gave polymers of similar performance as linear PNIPMAm but not as good as linear PNIPMAm with a very low-molecular weight polymer. Improvements to the KHI performance could possibly be made by judicial choice of the backbone from which to branch the VCap or NIPMAm chains.^46^ We are currently investigating this method with new backbones.
